# Pharmacokinetics and pharmacodynamics of ampreloxetine, a novel, selective norepinephrine reuptake inhibitor, in symptomatic neurogenic orthostatic hypotension

**DOI:** 10.1007/s10286-021-00800-x

**Published:** 2021-03-29

**Authors:** Arthur Lo, Lucy Norcliffe-Kaufmann, Ross Vickery, David Bourdet, Jitendra Kanodia

**Affiliations:** 1 Clinical and Translational Pharmacology, Theravance Biopharma US, Inc., 901 Gateway Boulevard, South San Francisco, CA 94080 USA; 2 Theravance Biopharma Ireland Limited, Dublin, Ireland; 3 Clinical Science, Neurology, Theravance Biopharma US, Inc., 901 Gateway Boulevard, South San Francisco, CA 94080 USA

**Keywords:** Ampreloxetine, Norepinephrine reuptake inhibitor, Neurogenic orthostatic hypotension, Autonomic failure, Pharmacokinetics, Pharmacology

## Abstract

**Purpose:**

Ampreloxetine is a novel, selective, long-acting norepinephrine reuptake (NET) inhibitor being investigated as a once-daily oral treatment for symptomatic neurogenic orthostatic hypotension (*n*OH) in patients with autonomic synucleinopathies. The purpose of this study was to characterize the pharmacokinetic and pharmacodynamic profiles of ampreloxetine in this target population.

**Methods:**

Patients with *n*OH were enrolled in a multicenter, phase II clinical trial of ampreloxetine (NCT02705755). They received escalating doses over 5 days in the clinical research unit, followed by 20 weeks of open-label treatment and then a 4-week withdrawal. As neurochemical biomarkers of NET inhibition, we assayed plasma concentrations of norepinephrine (NE) and its main intraneuronal metabolite 3,4-dihydroxyphenylglycol (DHPG) pre- and post-ampreloxetine.

**Results:**

Thirty-four patients with *n*OH were enrolled. Plasma ampreloxetine concentrations increased with repeated escalating doses, with peak concentrations observed 6–9 h post-drug administration. The median ampreloxetine dose in the 20-week treatment phase was 10 mg once daily. Plasma ampreloxetine concentrations reached steady state by 2 weeks, with stable plasma levels over 24 h. No influence of age or renal function on ampreloxetine plasma concentrations was observed. On treatment, compared to baseline, plasma NE significantly increased by 71% (*p* < 0.005), plasma DHPG significantly declined by 22% (*p* < 0.05), and the NE:DHPG ratio significantly increased (*p* < 0.001).

**Conclusions:**

Persistent elevation of plasma NE levels accompanied by reduced DHPG levels after ampreloxetine suggests reduced neuronal reuptake and metabolism of NE in postganglionic efferent sympathetic neurons. The findings are consistent with long-lasting NET inhibition, which may increase vasoconstrictor tone, supporting once-daily ampreloxetine dosing in patients with *n*OH.

## Introduction

Standing lowers venous return, triggering a baroreflex-mediated increase in sympathetic nerve activity to the vasculature and the release of norepinephrine (NE) from sympathetic varicosities on postganglionic fibers innervating the vessels [1, 2]. Once NE is released, it diffuses from the adventitial layer of the vessel to act on α1-adrenergic receptors expressed on smooth muscle cells within the walls of the arteries, arterioles and small veins [2–4]. This modulates vascular smooth muscle tone, raises peripheral resistance by narrowing of the vessel lumen, and offsets the fall in cardiac output to maintain blood pressure [1, 5, 6].

The action of NE at the neuroeffector junction produces a long-lasting vasoconstriction [7, 8]. Therefore, to prevent blood pressure from rising excessively, up to 90% of the NE released is inactivated by its rapid removal and reuptake back into the sympathetic varicosities by the neuronal NE transporter (NET) [8–10]. After NET reuptake, NE is either recycled for re-release or metabolized within the neuron by monoamine oxidase and aldehyde reductase to 3,4-dihydroxyphenylglycol (DHPG) [11]. DHPG diffuses rapidly across the cell membrane into the extracellular fluid or into the bloodstream, where it can be measured in plasma [12]. The combined measurement of plasma NE and DHPG provides information about peripheral sympathetic function and NET activity. Diminished NET reuptake increases plasma NE levels and decreases plasma DHPG levels [12–19].

Neurogenic orthostatic hypotension (*n*OH) is a disorder of reduced NE release from postganglionic sympathetic neurons innervating the peripheral vasculature. It is the hallmark feature of neurological disorders that affect the sympathetic nervous system. After diabetic neuropathy, the most common causes of *n*OH are the autonomic synucleinopathies, which are a group of overlapping neurodegenerative disorders caused by the abnormal accumulation of misfolded alpha-synuclein protein at various levels within the efferent autonomic pathway [1, 5]. The synucleinopathies are associated with both preganglionic (multiple system atrophy [MSA]) and postganglionic patterns of neuronal cell loss (notably, Parkinson disease [PD], pure autonomic failure [PAF] and dementia with Lewy bodies [DLB]) [1]. Regardless of the location of the lesion, patients with synucleinopathies can fail to release NE appropriately, resulting in inadequate vasoconstriction and a fall in blood pressure on standing. If blood pressure falls below the lower limit to maintain cerebral perfusion, patients suffer from debilitating symptoms of lightheadedness, dizziness, or feeling faint, which can lead to syncope and falls [20, 21].

NET inhibitors are being developed as a potential therapy for patients with *n*OH. By decreasing the reuptake of NE after its release, they prolong its vasoconstrictor effect, resulting in a pressor response on standing in patients with autonomic failure. By enhancing residual sympathetic function, NET inhibition provides an alternative pharmacological approach to midodrine and droxidopa.

Ampreloxetine (TD-9855) is a novel, investigational, selective NET inhibitor. Its > 24-h half-life allows once-daily dosing, which may be particularly helpful in the neurodegenerative synucleinopathies, as patients develop varying degrees of dysphagia or cognitive dysfunction leading to poor medication compliance with multiple daily dosing regimens [22–24]. Previous studies in healthy adults and patients with intact autonomic reflexes (fibromyalgia, attention-deficit/hyperactivity disorders) show that oral ampreloxetine has a plasma half-life of 30–40 h, stable plasma levels over 24 h, and maximal NET occupancy at the 10-mg dose [23]. It is not known whether the pharmacokinetic and pharmacodynamic profiles of ampreloxetine are similar in patients with *n*OH due to autonomic synucleinopathies, who in addition to having an autonomic lesion, are also older and are known to have varying degrees of renal impairment [25].

The purpose of our study was to understand the pharmacokinetics and pharmacodynamics of ampreloxetine in a representative target population of patients with *n*OH caused by MSA, PD, or PAF. To understand the impact of ampreloxetine on sympathetic (adrenergic) function, we measured plasma concentrations of NE and its main intraneuronal metabolite DHPG.

## Methods

### Study population

Patients with *n*OH were recruited to participate in the study. The diagnosis of autonomic failure was confirmed with a standard battery of autonomic function tests (Valsalva, deep breathing, passive upright unmedicated head-up tilt), performed with beat-to-beat blood pressure and heart rate monitoring. The main inclusion criteria included (i) a severe fall in systolic blood pressure > 30 mmHg within 3 min of standing, (ii) an associated diagnosis of MSA, PD, or PAF fulfilling current consensus criteria [5, 26, 27], and (iii) sympathetic autonomic failure diagnosed by absent phase IV blood pressure overshoot after release of the Valsalva strain. The main exclusion criteria included (i) autonomic neuropathy as a result of diabetes, amyloidosis, autoimmune or toxic cause, (ii) use of other pressor agents (e.g., droxidopa, midodrine, ephedrine), (iii) use of antihypertensive medications for the treatment of essential hypertension, and (iv) concurrent medical illnesses including cardiac insufficiency or major depression. The study was registered at ClinicalTrials.gov (NCT# 02705755) and was conducted in accordance with the Declaration of Helsinki [28]. Informed consent was obtained in all cases. Local ethical approval was obtained at all sites, prior to study initiation.

### Study design

After the diagnosis of autonomic failure was made and the underlying synucleinopathy diagnosis (MSA, PD, PAF) confirmed on neurological examination, subjects were admitted to the clinical research center at participating sites. Fludrocortisone at a stable dose of 0.1 mg/day was permitted. Other pressor agents were discontinued and washed out at least 5 half-lives before admission to the clinical research unit. As inpatients, subjects were given a standardized low-carbohydrate diet and were instructed to maintain a stable fluid intake (1.5–2 L/day). Blood pressure was measured at heart level with automated (or manual) sphygmomanometer arm cuff while the subject was (i) supine elevated to 30° (at minute 5 and minute 10), (ii) standing immobile (at minutes 1, 3, 5 and 10), and (iii) in the seated position (at minute 5 and minute 10).

### Dose escalation study

Subjects were first enrolled in a single-blind dose escalation study where they received ascending oral doses of ampreloxetine while admitted to the clinical research unit from day –2 to day 6 (Part A). Study drug was administered before breakfast at the same time each day (e.g., 8 AM). On day 1, all subjects received placebo. On days 2–5, the first group of patients received 1 mg, 2.5 mg, 5 mg and 10 mg ampreloxetine, respectively, at ~ 8 AM on each day. Following a protocol amendment, the second group of patients received 2.5 mg, 5 mg, 10 mg and 20 mg ampreloxetine on days 2–5, respectively. Stopping criteria included safety concerns, intolerable side effects or seated blood pressure > 180/110 mmHg. Orthostatic vitals were obtained pre-dose and at 4, 7, 9 and 12 h post-dose.

### Open-label treatment study

Subjects with an ampreloxetine-induced pressor response (> 10 mmHg increase in seated systolic BP) and an improvement of > 1 point or more on the OH-symptom assessment of dizziness/lightheadedness in Part A proceeded into a washout phase (minimum 8 days) and were then enrolled in a 20-week, open-label, outpatient treatment phase (Part C). Ampreloxetine was dosed orally, once a day. Dose increases up to a maximum dose of 20 mg per day were permitted within the first month, with 7 days between dose increases. Orthostatic vitals were obtained pre-dose and at 4 h post-dose. At the end of the 20-week treatment period, subjects underwent a 4-week withdrawal to determine whether they worsened back to baseline.

### Pharmacokinetic and pharmacodynamic (PK–PD) sampling

In the dose escalation study, blood samples for both PK and PD were collected after 10 min in the seated position and were placed in a wet ice bath, centrifuged, and then frozen within 90 min. Samples were drawn pre-dose and 6–9 h post-dose on days 1, 3 and 5, and 24 h after the last administered dose. The 6–9-h time point was selected to coincide with the peak plasma ampreloxetine levels observed in prior studies with healthy volunteers [23].

In the open-label 20-week treatment study, PK blood samples were collected 6–9 h and 24 h post-drug administration on day 1. Additional samples were collected upon return to the clinic at the end of week 2 (15 days), week 4 (29 days), week 12 (85 days), and in the washout week 22 (day 155). Plasma concentrations of NE and DHPG were assayed pre-dose on day 1, and 6–9 h post-dose on day 29. Plasma ampreloxetine and DHPG concentrations were measured using liquid chromatography with tandem mass spectrometry detection (Q2 Solutions, Ithaca, NY, USA) and NE concentrations in Part C were measured with high-performance liquid chromatography (ARUP Laboratories, Salt Lake City, UT, USA). The lower limit of quantitation was 0.0500 ng/mL for ampreloxetine, 0.0400 ng/mL for DHPG, and 0.025 ng/mL for NE.

### Population PK–PD and statistical analysis

The pharmacokinetic analysis data set included all patients with ≥ 1 plasma ampreloxetine concentration measurement. Ampreloxetine exposure (AUC, area under the concentration–time curve from 0 to 24 h) was individually estimated for each patient from the sparsely sampled plasma concentrations, using the a model-specific approach previously described for ampreloxetine from a population pharmacokinetic analysis [23]. AUC was estimated for each day of dosing in Part A, and steady-state AUC was estimated for Part C.

The pharmacodynamic analysis set included all patients with ≥ 1 evaluable NE and DHPG concentration. For calculation of time-matched change in DHPG, the two plasma samples collected on day 1 (placebo) in Part A and the single pre-dose plasma sample in Part C were averaged to estimate baseline values. The relationship between plasma concentration of ampreloxetine and DHPG (calculated as change from time-matched baseline) in Part A was fitted to a standard *E*_max_ (drug concentration producing maximum effect) relationship. In Part C, the statistical significance of change in NE concentration, DHPG concentration, and the ratio of NE concentration to DHPG concentration (NE:DHPG ratio) was calculated using a paired *t* test for geometric mean ratios for subjects pre-dose and on day 29. Pre-dose supine systolic blood pressure (SBP) measurements on day 1 of Part A and Part C were used as baseline measurements for the calculation of change from baseline.

Renal function was determined using the estimated glomerular filtration rate (eGFR) calculated using the Modification of Diet in Renal Disease equation [29, 30]. The influence of age and renal function on predicted steady-state AUC was tested using a linear regression model where the significance of the relationship was determined using the *t* test. Analysis was performed with Prism 8 software (GraphPad Software, San Diego, CA, USA). All data are mean ± standard deviation, unless otherwise stated. Significance was set at *p* < 0.05.

## Results

### Patient demographics

Thirty-four patients (MSA *n* = 18, PAF *n* = 7, PD *n* = 9) were enrolled in the study (day 1, Part A). Thirty-one patients had at least one pharmacokinetic sample available pre- and post-ampreloxetine, and 29 patients had samples available at the end of the titration 24 h after their maximum tolerated dose (day 5, Part A). Twenty-one patients (MSA *n* = 12, PAF *n* = 4, PD *n* = 5) were enrolled in the open-label 20-week treatment phase and had at least one sample available while on steady-state treatment. Sixteen patients had samples available after 4 weeks on ampreloxetine (day 29).

The age range of the patients in the phase II trial was 51–83 years, and their average body mass index was 25.9 ± 4.04 kg/m^2^. As expected in chronic autonomic failure patients [25], eGFR on average was mildly reduced at 72.0 ± 17.7 mL/min/1.73 m^2^ (range 25.5–113 mL/min/1.73 m^2^), with seven patients (21%) having moderate renal impairment (eGFR ≤ 59 mL/min/1.73 m^2^). There was a slight predominance of men with available pharmacokinetic data in Part A (20 men, 11 women), but similar numbers of men and women in Part C (12 men, 9 women).

### Pharmacokinetics of ampreloxetine

In the dose escalation study (Part A), ampreloxetine exposure at 6–9 h post-dose increased with escalating doses up to 20 mg ampreloxetine (Table 1). Furthermore, the plasma concentration of ampreloxetine at 24 h after the last dose was ~ 75% of the corresponding plasma concentration at 6–9 h after the last dose. The concentration profile observed in patients with autonomic failure was consistent with a sustained plasma ampreloxetine concentration profile over the 24-h dosing interval and similar to the profile observed in subjects with intact autonomic reflexes [23].

**Table 1:** Summary of ampreloxetine concentration (ng/mL) over time

	6–8 or 6–9 h post-dose				24 h post-dose	
	2.5 mg^a^	5 mg^a^	10 mg^b^	20 mg^b^	10 mg^c^	20 mg^c^
*n*	15	14	11	12	15	13
Mean	1.37	3.09	6.95	13.2	5.23	9.79
SD	0.556	1.84	3.04	4.61	2.07	4.84
(Min, Max)	0.557–2.51	1.19–8.51	2.73–12.7	5.27–22.6	2.38–8.93	3.04–17.9
Median	1.35	2.59	6.34	13.0	5.19	8.94
Max	2.51	8.51	12.7	22.6	8.93	17.9

Subjects received 1–10 mg or 2.5–20 mg ampreloxetine once daily on days 2–5

^a^Ampreloxetine plasma concentration measured on day 3

^b^Ampreloxetine plasma concentration measured on day 5

^c^Ampreloxetine plasma concentration measured on day 6

**Fig. 1:**
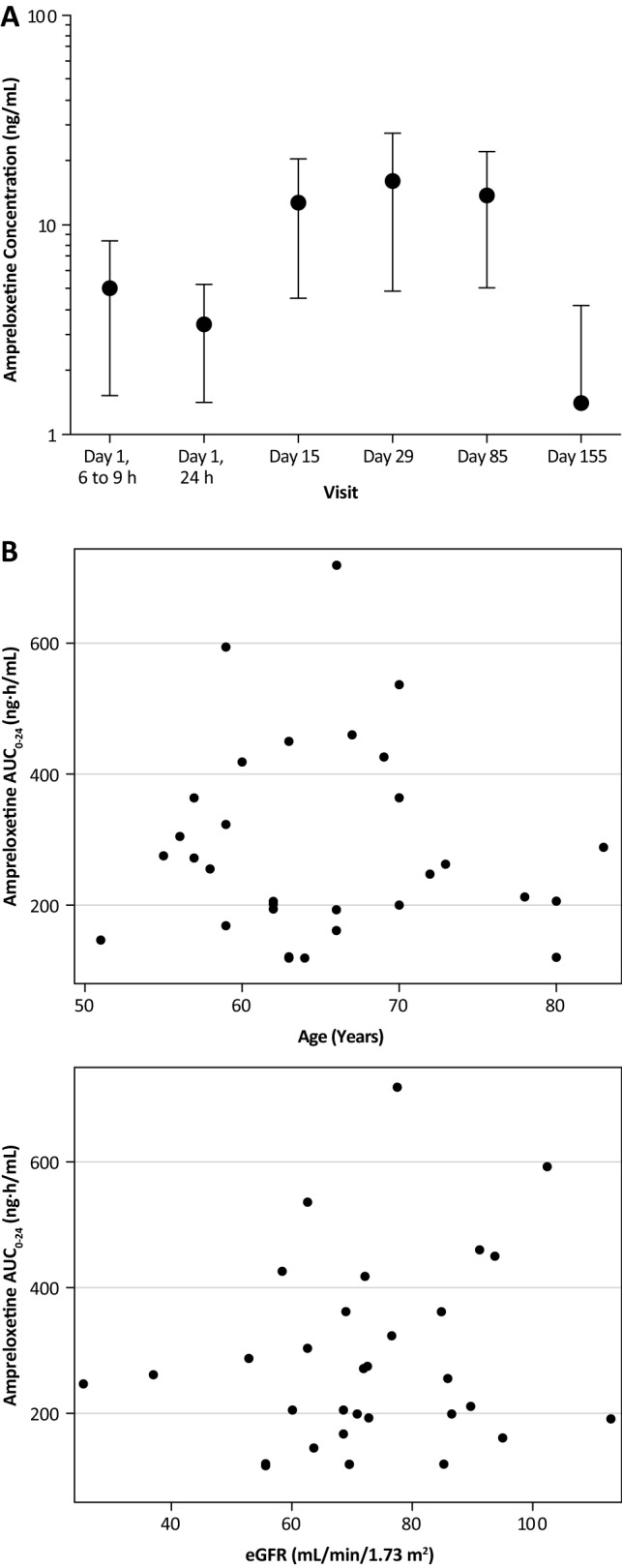
Ampreloxetine concentration and exposure: **a** mean ± SD plasma ampreloxetine concentrations (ng/mL) following daily oral administration of ampreloxetine for up to 20 weeks (Part C; dose levels combined for each sampling day/time—semi-log scale). Day 140 was the last day of dosing. Plasma concentrations of ampreloxetine (some below the limit of quantitation) were detected at week 22 (day 155) owing to incomplete washout. **b** Scatter plot of the individually predicted ampreloxetine steady-state exposures (area under the concentration–time curve from 0 to 24 h [AUC_0–24_]) vs. age and renal function (estimated glomerular filtration rate [eGFR]) of all patients

In the 20-week open-label treatment phase (Part C), the median dose was 10 mg once daily. Steady-state plasma levels of ampreloxetine were achieved before week 2 (on day 15), with similar plasma concentrations observed at the end of week 2 (day 15; 12.7 ± 8.2 ng/mL), week 4 (day 29; 16.1 ± 11.3 ng/mL), and week 12 (day 85; 13.6 ± 8.6 ng/mL) (Fig. 1a). Interindividual variability ranged from 57% (day 1, 24 h post-dose) to 70% (week 4 [day 29]). After withdrawal, as was expected, the plasma concentration of ampreloxetine decreased tenfold to 1.39 ± 2.7 ng/mL on week 22 (day 155), 2 weeks after the last dose.

Ampreloxetine exposure in subjects with *n*OH was consistent with previously published reports used as a basis for population pharmacokinetic modeling [23]. No association was detected between age (51–83 years, *β* = −1.01, *t*(29) = −0.291, *p* = 0.773) or renal function (eGFR = 26–113 mL/min/1.73 m^2^_,_ *β* = 1.45, *t*(29) = 0.972, *p* = 0.339) with the model-estimated steady-state ampreloxetine exposure (AUC) (Fig. 1b). Population PK analysis conducted using the approach outlined in Kanodia et al. [23] indicated that ampreloxetine exposure in patients with *n*OH was comparable to exposure in all historical studies, and that the pharmacokinetics of ampreloxetine was independent of disease status.

### Pharmacodynamics

**Fig. 2:**
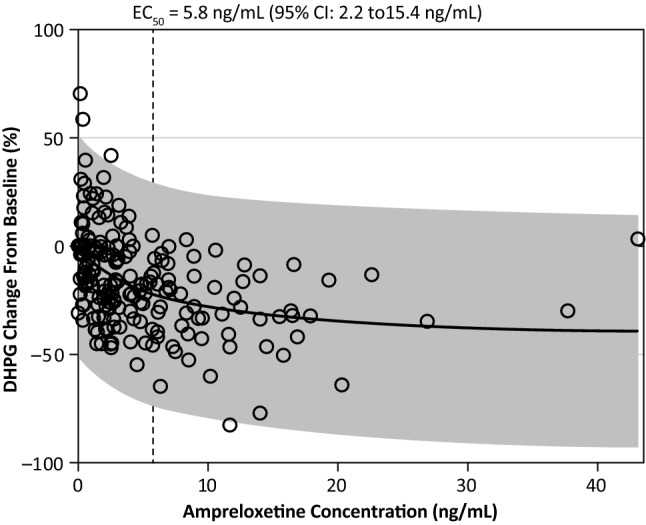
Pharmacokinetics of ampreloxetine: scatter plot of the change from time-matched baseline plasma DHPG vs. ampreloxetine concentrations with corresponding fit to maximum effect (*E*_max_) relationship. *DHPG* 3,4-dihydroxyphenylglycol

In the dose escalation study (Part A), data from a total of 31 patients (206 total data points) with available time-matched measurements of plasma ampreloxetine and DHPG concentrations were evaluated to explore the pharmacokinetic–pharmacodynamic relationship. The time-matched change from baseline in plasma DHPG decreased with increasing plasma concentrations of ampreloxetine, with a fitted half-maximal inhibitory concentration value of 5.8 ng/mL (95% CI 2.2–15.4 ng/mL) (Fig. 2).

**Fig. 3:**
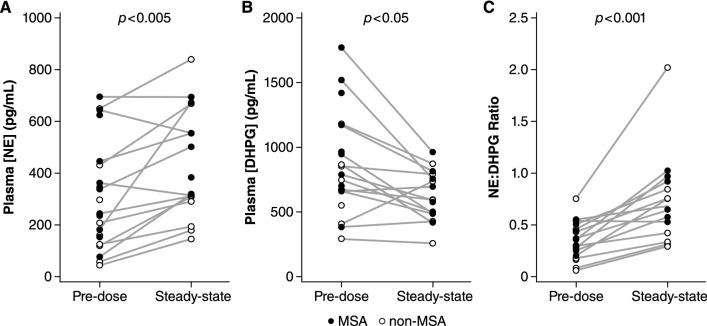
Pharmacodynamics of ampreloxetine: **a** Shows significant increase in plasma NE concentration post-ampreloxetine. **b** Shows significant decrease in plasma DHPG concentrations post-ampreloxetine. **c** Shows increase in the ratio of NE to DHPG concentrations. All data are pre-dose and after 4 weeks of open-label treatment at a median dose of 10 mg per day (day 29). *NE* norepinephrine, *DHPG* 3,4-dihydroxyphenylglycol. Statistical significance was determined with a ratio paired *t* test

In the open-label treatment phase (Part C), the pharmacodynamic response at week 4 was expected to be representative of the steady-state response. As shown in Fig. 3, the geometric mean ratio (GMR) of plasma NE concentrations on day 29 relative to pre-dose baseline was 1.71 (95% CI 1.28–2.29, *p* < 0.005), and the corresponding geometric mean ratio of plasma DHPG concentrations was 0.775 (95% CI 0.636–0.932, *p* < 0.050). These responses are consistent with NET inhibition and a reduction in intraneuronal NE metabolism, and resulted in a significant increase in the NE:DHPG ratio after 4 weeks of ampreloxetine treatment (GMR 2.21, 95% CI 1.71–2.84, *p* < 0.001). The increase in the NE:DHPG ratio was similar in MSA and non-MSA subjects and remained statistically significant in each subgroup (MSA *p* < 0.005, non-MSA *p* < 0.005).

**Fig. 4:**
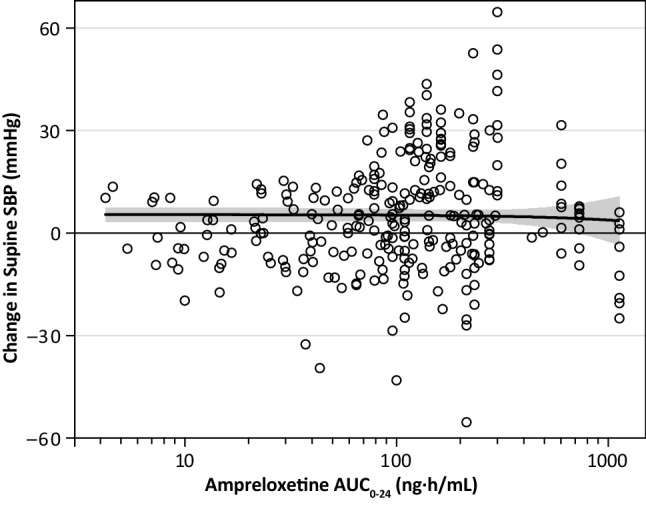
Effect on supine blood pressure. Scatter plot of the change from baseline in supine systolic blood pressure vs. individually predicted ampreloxetine exposures (AUC_0–24_), with corresponding linear fit showing no relationship. AUC_0–24_, area under the concentration–time curve from 0 to 24 h. *DHPG* 3,4-dihydroxyphenylglycol, *SBP* systolic blood pressure

Data from 31 patients in Part A and Part C (281 total measurements) were used to determine the exposure–response relationship between the individually predicted population AUC and the time-matched change in supine SBP from baseline (*β* = −0.0016, *t*(279) = −0.36, *p* = 0.719). No significant correlation between supine SBP and ampreloxetine exposure was observed with once-daily ampreloxetine administration (Fig. 4).

## Discussion

The study demonstrates for the first time that ampreloxetine, at a median dose of 10 mg per day, significantly inhibited peripheral NET activity in patients with autonomic failure. This was shown by an increase in the concentration of NE and a decrease in the concentration of DHPG in plasma, reflecting reduced NE metabolism in peripheral sympathetic neurons. Importantly, ampreloxetine produced a steady state of NET inhibition with a terminal half-life of 30–40 h.

The pharmacokinetic profile of ampreloxetine in patients with autonomic failure was consistent with that observed in healthy volunteers and patient groups with intact cardiovascular autonomic reflexes [23]. The similarly low ratio of peak to trough plasma ampreloxetine concentrations (Table 1) observed over the 24-h period following oral dosing in patients with autonomic failure makes it potentially suitable as a once-daily therapy for the treatment of *n*OH. This would be the first pressor agent that did not require multiple daily dosing. Encouragingly, we saw no impact of age or compromised renal function on the pharmacokinetics and pharmacodynamics of ampreloxetine, which is important, as *n*OH has a variable age of onset (beginning the fifth to sixth or seventh decade of life) and is associated with target organ damage in the kidney owing to supine hypertension [25, 31]. It is well known that treatment of *n*OH with pressor agents can be hampered by the exacerbation of supine hypertension [32]. Another reassuring observation is the lack of a clear correlation between plasma ampreloxetine concentrations and supine SBP. While the results of the hemodynamic effects are still being examined, there was no consistent signal for a worsening of supine hypertension with increasing ampreloxetine levels.

The increase in plasma NE concentrations post-ampreloxetine suggests that the selective NET inhibitor could increase blood pressure by raising peripheral vascular resistance. This response to NET inhibition is likely to be enhanced and have therapeutic potential in patients with autonomic failure, who lack the central sympatholytic mechanisms that would normally buffer any pressor effect [33]. The decrease in plasma DHPG concentrations post-ampreloxetine suggests that the NE reuptake after its release is blocked. The major fraction of NE taken back up by the postganglionic sympathetic neurons is inactivated by recycling into vesicles or metabolism ending in DHPG. The significant reduction in DHPG levels observed within 2 weeks and its sustained reduction over the long-term open-label treatment phase suggests that ampreloxetine may be a durable treatment for patients with *n*OH. A comprehensive manuscript describing the efficacy analysis with the blood pressure and symptom response observed throughout the phase II trial, adverse events, and dropouts is in preparation. In the open-label extension trial (Part C), at the 10-mg dose, plasma NE levels increased almost twofold, which is in proportion with the physiologically normal response to standing in which NE levels are expected to double from supine to standing [1, 34]. These results support use of the 10-mg dose in the ongoing double-blind and open-label phase III studies (NCT03750552, NCT03829657, NCT04095793).

One intriguing question is whether the pharmacodynamic response differs according to the underlying diagnosis. A side effect of NET inhibition in patients with intact autonomic function is hypotension, due to centrally mediated sympatho-inhibition [35] at the level of rostral ventrolateral medulla alpha_2_-adrenoceptors [36]. However, in patients with lesions within the efferent sympathetic pathways, the reverse effect is observed, as the peripheral effects of increasing vasoconstriction dominate, producing an increase in blood pressure [33]. The magnitude of the pressor response in response to NET inhibition in patients with efferent autonomic failure likely depends on the degree of remaining postganglionic sympathetic neurons [33]. It is tempting to speculate that because MSA is more likely to be associated with a preganglionic lesion, the pressor response induced by ampreloxetine would be larger than in patients with Lewy body disorders that are thought to have a greater extent of postganglionic sympathetic neuronal loss, as was observed with atomoxetine [33]. However, approximately one-third of patients with MSA have imaging and postmortem findings consistent with postganglionic sympathetic denervation of the heart [37, 38]. Likewise, there are patients with Lewy body forms of synucleinopathies (PD/DLB) that have *n*OH with normal levels of plasma NE. This considerable overlap in NE levels between Lewy body disorders and MSA suggests that the autonomic phenotypes can be quite mixed [1, 34]. In addition, prospective observational studies show that patients diagnosed with PAF may phenoconvert to MSA, DLB or PD [39]. Thus, rather than the diagnosis per se, it may be the extent of remaining postganglionic sympathetic fibers and degree of denervation sensitivity that predicts the response to pressor agents [40]. This concept is being further explored in the phase III clinical program of ampreloxetine.

There are some limitations to our study. First, the sample size was too small to reliably detect differences in the pharmacodynamic response due to the diagnosis or predominant site of the lesion. Plasma levels of DHPG and NE may not entirely reflect what is occurring at the neurovascular effector junction given the complex dynamic release, reuptake and intraneuronal metabolism and its systemic clearance [6, 11, 13–19, 41]. Nevertheless, we controlled for diurnal variability by taking samples at the same time of day and controlled for postural changes by always drawing the blood in the semi-supine position. Another limitation of the study is that we measured NE and DHPG levels at rest in the seated position at a single time point and did not repeat measures during increases in sympathetic outflow, for example during standing, which would provide a more accurate means to access the extent of norepinephrine re-uptake inhibition [16, 17, 19]. Finally, the study design does not exclude the possibility that the observed decrease in plasma DHPG may reflect a decrease in vesicular leakage rather than blockade of reuptake.

In summary, the pharmacokinetic and pharmacodynamic profiles of ampreloxetine in patients with *n*OH due to synucleinopathies support the concept that this pharmacological approach could be helpful in this population. The observed increase in plasma NE, combined with a decrease in DHPG, observed 9 h post-ampreloxetine supports the mechanism of action of long-acting blockade of the NET. Based on the results of this study, ongoing phase III studies will determine the safety, efficacy and durability of 10 mg once-daily ampreloxetine for the treatment of symptomatic *n*OH in patients with autonomic synucleinopathies.

## Data Availability

Not applicable.

## Declarations

### Availability of data and material

Theravance Biopharma US, Inc. (and its affiliates) will not be sharing individual de-identified participant data or other relevant study documents.
